# Patterns of free amino acids in tundra soils reflect mycorrhizal type, shrubification, and warming

**DOI:** 10.1007/s00572-022-01075-4

**Published:** 2022-03-21

**Authors:** Louise C. Andresen, Samuel Bodé, Robert G. Björk, Anders Michelsen, Rien Aerts, Pascal Boeckx, J. Hans C. Cornelissen, Kari Klanderud, Richard S. P. van Logtestijn, Tobias Rütting

**Affiliations:** 1 Department of Earth Science, University of Gothenburg, Gothenburg, Sweden; 2 Isotope Bioscience Laboratory (ISOFYS), Department of Green Chemistry and Technology, Ghent University, Ghent, Belgium; 3 Gothenburg Global Biodiversity Centre, Gothenburg, Sweden; 4 University of Copenhagen, Copenhagen, Denmark; 5 Department of Ecological Science, Vrije Universiteit Amsterdam, Amsterdam, The Netherlands; 6 Faculty of Environmental Sciences and Natural Resource Management, Norwegian University of Life Sciences, Aas, Norway

**Keywords:** Tundra, Global warming, Ericoid mycorrhiza, Ectomycorrhizal plants, Amino acid uptake, Nitrogen cycling

## Abstract

The soil nitrogen (N) cycle in cold terrestrial ecosystems is slow and organically bound N is an important source of N for plants in these ecosystems. Many plant species can take up free amino acids from these infertile soils, either directly or indirectly via their mycorrhizal fungi. We hypothesized that plant community changes and local plant community differences will alter the soil free amino acid pool and composition; and that long-term warming could enhance this effect. To test this, we studied the composition of extractable free amino acids at five separate heath, meadow, and bog locations in subarctic and alpine Scandinavia, with long-term (13 to 24 years) warming manipulations. The plant communities all included a mixture of ecto-, ericoid-, and arbuscular mycorrhizal plant species. Vegetation dominated by grasses and forbs with arbuscular and non-mycorrhizal associations showed highest soil free amino acid content, distinguishing them from the sites dominated by shrubs with ecto- and ericoid-mycorrhizal associations. Warming increased shrub and decreased moss cover at two sites, and by using redundancy analysis, we found that altered soil free amino acid composition was related to this plant cover change. From this, we conclude that the mycorrhizal type is important in controlling soil N cycling and that expansion of shrubs with ectomycorrhiza (and to some extent ericoid mycorrhiza) can help retain N within the ecosystems by tightening the N cycle.

**Supplementary information:**

The online version contains supplementary material available at 10.1007/s00572-022-01075-4.

## Introduction

Mycorrhizal fungi assist plants in acquiring nutrients (Newsham et al. 2009; Hewitt et al. 2020). Currently, the Arctic is undergoing vegetation changes including “shrubification,” i.e., shrub expansion, as a consequence of global warming (Martin et al. 2017; Bjorkman et al. 2018; Myers-Smith et al. 2019). With changing plant species dominance, we also expect a change in mycorrhizal association (Lorberau et al. 2017; Zhao et al. 2018; Volwes and Björk 2019). Changes in the plant community can have strong effects on the soil carbon (C) and nitrogen (N) cycle (Schimel and Chapin III 1996, Hicks et al. 2020). It is known that in N poor ecosystems, such as alpine and arctic tundra, plants with their mycorrhizal fungal associates can directly take up free amino acids (fAA) and other monomeric N compounds, bypassing the mineralization to inorganic N, and thereby shortcutting the N cycle (Schimel and Bennett 2004). Depending on the type of mycorrhizal association, plants can access N bound in soil organic matter (SOM) (Read and Perez-Moreno 2003; Clemmensen et al. 2013). Ecosystems dominated by ectomycorrhizal (ECM) fungal communities are characterized by relatively slow soil nutrient cycling with only little loss of inorganic nutrients, in contrast to arbuscular mycorrhizal (AM)-supported vegetation types with rapid nutrient cycling and substantial nitrification (Phillips et al. 2013). Ecosystem nutrient cycling rates are mainly driven by litter decomposition rates. The ECM- and the AM-dominated ecosystems have different nutrient turnover rates, as litter from ECM plant species in general has a relatively slow decomposition, whereas AM plant species exhibit faster litter decomposition (Cornelissen et al. 2001). Also, litter from plant species with ericoid mycorrhizal (ERM) fungi consistently have relatively poor decomposability (Cornelissen et al. 2001). Hence, there is a strong relation of the dominant plants and their mycorrhizal types with decomposition, controlled both by the litter quality and the abilities of the mycorrhizal fungi to access N. For example, ECM fungi have about twice as many genes related to N metabolism compared to AM fungi (Pellitier and Zak 2018).

Free amino acids reside in the soil for a limited time and are rapidly taken up by N demanding microorganisms (including mycorrhizal hyphae) and plant roots (Jones and Kielland 2012; Homyak et al. 2021). Free-living soil microorganisms acquire intact fAA rather as a C source than as an N source (Soong et al. 2020). Plants with ECM associations utilize both organic (such as fAA) and inorganic N resources whereas non-mycorrhizal (NM) and AM plants rely more on inorganic N sources (Peay 2016). Hence, the use of soil N resources in a plant community depends on the dominant plant species and their associated mycorrhizal types. Theoretically, ECM plants are more competitive than AM plants when soil has a high stock of organic N (Peay 2016). The fAA content in soil is controlled directly by the balance between fAA production (mainly hydrolyzation of AA polymers), leading to the inflow of amino acids to the soil (Wanek et al. 2010; Andresen et al. 2016a), and by plant and microbial uptake (consumption) (Näsholm et al. 2009). Plants also affect the fAA composition directly by root exudation (Moreau et al. 2019). The relative effectivity of the fAA-N uptake in tundra ecosystems has been investigated using stable isotope (^15^N and ^13^C) tracing. There are no general conclusions on dominant mycorrhizal type in organic N uptake as yet, but significant differences in plant uptake of fAA between types of mycorrhizal associations were found in subarctic heaths, suggesting proportionally higher uptake of fAA by ECM and ERM fungi than by AM fungi and NM types (Andresen et al. 2008; Ravn et al. 2017). ERM fungi are capable of acquiring N from peptides and amino acids (Bajwa and Read 1985, Sokolovski et al. 2002), and attack structural polymers of SOM to access organic N (Read and Perez-Moreno 2003; Dynarski and Houlton 2020). The ERM symbiosis is therefore often viewed as similarly efficient in acquiring N from SOM as the ECM symbiosis (Dynarski and Houlton 2020), but ECM fungi may differ widely in their capacity to break down organic matter (Clemmensen et al. 2021). Hence, vegetation composition can, through its association with mycorrhizal fungi, have a strong impact on soil chemistry, because plant functional group composition and soil fAA content are related (Yano et al. 2013).

Arctic and subarctic ecosystems generally have a slow turnover of litter and SOM because of low temperatures restricting decomposition, low chemical litter quality, and constraints from saturated or very low water availability (Pascual et al. 2021). These soils are vulnerable to global warming because an increase in soil temperature can accelerate SOM decomposition (Cornelissen et al. 2007) and provide access to organic N in deeper soil layers through permafrost thaw (Hewitt et al. 2020; Pedersen et al. 2020), leading to stimulated N turnover (Keuper et al. 2012). Consequently, soil C and SOM stocks can decrease (Wieder et al. 2019; Jung et al. 2020), and with faster enzymatic processes (Brzostek et al. 2012), depolymerization rates will increase, thereby potentially increasing fAA pools in soils under warming. However, increased plant or microbial uptake of fAA can counteract this, and potentially lead to a decrease in soil fAA content (Andresen et al. 2009; Hicks et al. 2020).

We aimed to investigate if soil fAA content across tundra sites was associated with vegetation and mycorrhizal types, if warming affected the fAA content, and if the effect of warming depended on site. Thereto, we measured soil fAA contents in plots exposed to decade-long warming in five field experiments with Open Top Chambers (OTCs) in northern Scandinavia. We hypothesized that fAA content is lower in soils underneath vegetation with dominance of ECM and ERM fungal communities due to their efficient N uptake from organic N sources and their slower litter decomposition rates. We also hypothesized that fAA content should be reduced in sites where experimental warming has led to expansion of ECM and ERM shrubs compared to ambient conditions.

## Methods

### Study sites

The study sites (Table S1) were located in the subarctic (Latnajaure and Abisko) and alpine (Finse) regions in the Scandinavian Mountain range. All sites had long-term warming experiments with OTCs (Marion et al. 1997). The Latnajaure Field Station (68° 21′ N 18° 30′ E) is situated about 15 km west of Abisko in northern Sweden and resembles the low-arctic phytogeography (Björk et al. 2007a, b), with mesic meadow as one of the many vegetation types. The temperature ranged from the coldest month, February, average −9.7 °C, to the warmest month, July, average 8.6 °C during 1992–2019 (Scharn et al. 2021). Abisko Scientific Research Station (68° 21′ N 18° 49′ E) houses three OTC experiments. One is a blanket-bog at the shore of lake Torneträsk (Dorrepaal et al. 2009; Weedon et al. 2012). Second is at a wet heath near lake Torneträsk, and third at a mesic heath (Michelsen et al. 2012). The snow-free season usually lasts from late May to early October (Abisko Scientific Research Station Meteorological Station, 1986–2015 averages; Callaghan et al. 2010; Hicks et al. 2020). At Finse (60° 21′ N 7° 13′ E) in the northern part of Hardangervidda, Norway, an OTC experiment is situated in a dry heath (Klanderud and Totland 2007). Finse has a summer mean monthly temperature during June, July, and August of 6.3 °C (Klanderud 2008).

At each site, the OTCs and control plots were at least 2 m apart; the control plots were marked out by the corners. The effect of warming, the duration of the experiment, dominant vegetation type, height above sea level, and climatic information are given in Table S1.

### Plant and soil sampling

Data of plant cover percentage inside the OTC and control plots was obtained from databases (unpublished). This data was determined by using the point intercept method (Molau and Mølgård 1996) or by recording species abundances in subplots and calculating sub-plot frequencies (Klanderud 2008). Plant cover was classified by the plant functional types: forbs, graminoids, shrubs, and mosses; and by type of mycorrhizal association, such as ERM, ECM, and NM combined with AM groups. For classification of mycorrhizal type, we used the classifications described by Michelsen and co-authors (Michelsen et al. 1996, 1998), who investigated mycorrhizae in subarctic and arctic plants including Abisko sites nearby the current study sites. While the degree of mycorrhizal colonization may change over the year, it is unlikely that change in types of mycorrhizal colonisations may occur. The AM plants were grouped with NM plants, as some sites had no or very low presence of AM species, hereafter called AM/NM as one group. Furthermore, *Arctostaphylos* spp*.* was grouped functionally with ECM, as this genus shows Arbutoid mycorrhizal colonization morphology, which resembles the ECM symbiosis in mycorrhizal function (Michelsen et al. 1998). Plant cover data was assembled from databases from the year closest to the time of soil sampling in 2013: 2013 for blanket bog, 2011 for wet heath, 2014 for mesic heath, 2016 for mesic meadow, and 2011 for dry heath, as these communities are dominated by perennials the coverage varies little from year to year.

For soil sampling, the vegetation and moss-lichen crust was gently pushed aside. Soil was sampled with a knife or soil corer in the predominant rooting zone (at dry, wet, and mesic heath down to bedrock). Thus, we sampled from the surface down to 3–5 cm depth in dry heath, and bulked three subsamples. At the other sites, only one soil core was taken, at mesic meadow from surface down to 5 cm depth, in blanket bog to 12 cm depth, in wet heath to 3–8 cm depth and in mesic heath to 6–11 cm depth. The soils were kept cold, and upon returning to the field station laboratory, the samples were hand-sorted to remove roots, green plant material, and stones for a maximum of 10 min per sample. Fresh soil was extracted in a weight ratio 1 to 3 with 10 mmol$\cdot$L^−1^ CaSO_4_ solution containing 3.4% formaldehyde, shaken for 30 min, then filtrated (Whatman 1 qualitative filter papers 12.5 cm diam.) and shipped to ISOFYS (Belgium) as frozen extracts.

The CaSO_4_ extracts were purified using SPE (solid phase extraction) cation-exchange cartridges (OnGuard II H, 1 cc, Dionex). The SPE cartridges were conditioned with ultrapure water (> 18.2 MΩ), then 10 mL 3 mol L^−1^ NH_3_, 10 mL ultrapure water, and 10 mL 1 mol L^−1^ HCl, and finally 10 mL ultrapure water. After loading the extract on the cation-exchange resin, the cartridge was washed with 10 mL ultrapure water, and the purified amino acids were eluted with 30 mL 3 mol L^−1^ NH_3_. The purified sample was dried under reduced pressure at 35 °C, and finally derivatized using ethanol-pyridine and ethylchloroformate (Wanek et al. 2010).

### Analysis of amino acids with GC–MS

During purification, an internal amino acid standard was added to samples, which was a mixture of three non-biological amino acids: nor-valine, nor-leucine, and 4-chloro-phenylalanine. The method described by Wanek et al. (2010) was developed further at ISOFYS, Ghent University (Andresen et al. 2015). Concentrations of amino acids were determined using gas chromatography–mass spectrometry (GC–MS, Trace GC–DSQ, Thermo Fisher). Separation was done on a VF 5-MS column (30 m × 0.25 mm ID × 0.25 µm film). With the available technical capacity, we focused on 14 amino acids: alanine, glycine, valine, leucine, serine, isoleucine, threonine, proline, asparagine, aspartate, methionine, glutamate, phenylalanine, and tyrosine.

### Calculations and statistical analysis

In order to test overall effects of warming, site, and their interaction on soil properties, a multivariate ANOVA (MANOVA) using Wilks lambda was made for the 14 fAA content and other soil properties (SOM, dissolved organic C, microbial biomass C and N, NO_3_-N, NH_4_-N, and total fAA-N). The MANOVA test for overall effects of the factors on multiple related response variables, and in case of significance, the individual response variables were subsequently tested by two-way ANOVA for all soil properties, and for the plant cover summed by type of mycorrhizal associations and by plant growth forms. In addition, significant differences between sites (including both treatments) were tested by Tukey’s test. Within site, the effect of treatment was tested with one way ANOVA for each soil property. Effects of treatment from the tests were considered significant when *P* < 0.05 and denoted as a tendency when *P* < 0.1. We explored if data met the assumptions of ANOVA (variance homogeneity, normality) and log transformed the data if required. All analysis of variance was performed using SAS 9.4.

The redundancy analysis (RDA), in which the gradient lengths were 1.4 SD units, was conducted using CANOCO 5.04 to investigate the relationship between types of mycorrhizal associations and content of individual fAA across sites and treatments.

The “responsiveness” to warming for variables representing vegetation cover and soil properties at a site was expressed as logarithmic response ratio (LRR) calculated as follows (van Wijk et al. 2003; Andresen et al. 2016b): LRR = ln(warmed_av_/control_av_).

## Results

### Growth form and mycorrhizal types

Both graminoid and forb cover differed between sites (both *P* < 0.0001, ns for treatment and the interaction). Graminoid coverage ranged from a mean of 5% (4% std. dev.) at mesic heath warmed and 5 (2) % at blanket bog control, up to as much as 95 (17) % at wet heath warmed. Forbs ranged from 1 (1) % at blanket bog control to 42 (55) % at wet heath warmed. At the blanket bog, the graminoids tended to increase under warming, from 5 (2) to 19% (13) (*P* = 0.073).

Shrub and moss cover also differed significantly between sites with shrubs ranging from 21 (14) % at mesic meadow control to 212 (76) % at blanket bog control, and mosses ranged from 2 (2) % at mesic heath warmed to 100 (0) % at all blanket bog plots (both with *P* < 0.0001 for site). We also observed interactions between site and treatment (*P* = 0.032 for shrubs and < 0.0001 for mosses). Shrubs increased under warming at the mesic heath from 95 (14) to 152 (14) % (*P* = 0.003). Moss cover decreased under warming at the mesic meadow from 57 (17) to 28 (10) % (*P* = 0.004) and at the mesic heath from 26 (18) to 2 (2) % (*P* = 0.038).

At the dry heath, ECM plant cover decreased from 64 (26) in control to 53 (19) % in warmed plots (*P* = 0.042). At the mesic meadow, the AM/NM mycorrhizal type plant cover tended to decrease under warming (33 (14) to 22 (2) %, *P* = 0.086) (Table S2).

### Soil-free amino acids

**Fig. 1:**
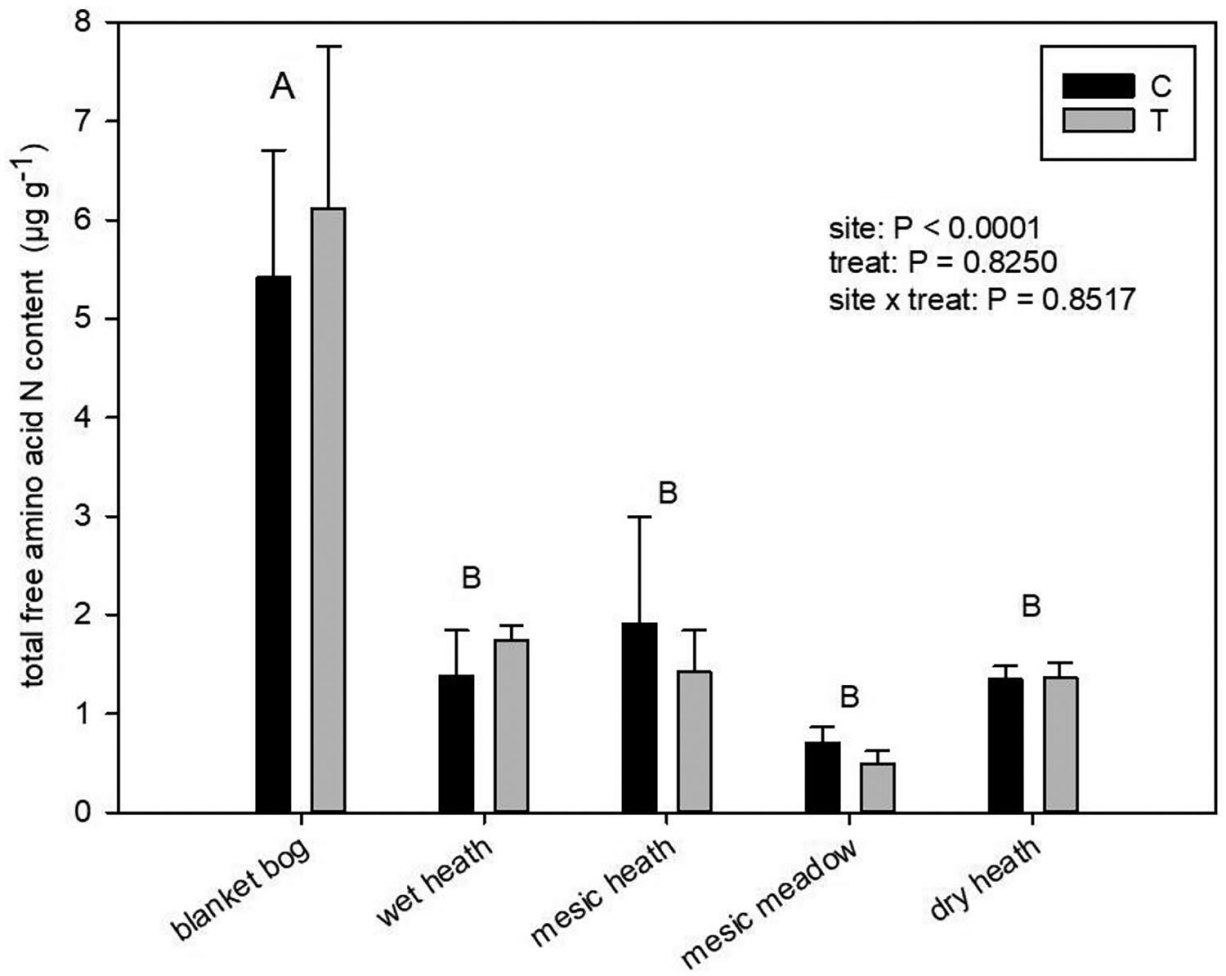
Total free amino acid nitrogen (fAA-N) content in soil from wet heath, mesic heath, mesic meadow, dry heath, and blanket bog, for control (C) and warming treatment by OTC (open top chamber, T). *P* values of the effect of site and warming treatment and their interaction. Different letters represent significant differences between sites

Total fAA-N varied 12-fold among sites and treatments with the blanket bog having higher total fAA-N compared to the other sites (*P* < 0.0001). There was no significant effect of treatment nor interaction between site and treatment (Fig. 1, Table S3). Similar results were found for fAA-C (site *P* < 0.0001, data not shown). Predominant amino acids in soil extracts were in general glutamate, aspartate, and alanine, with the exception of the mesic meadow where only glutamate dominated (Fig. S1). Additional predominant amino acids were glycine at the mesic heath and serine at the blanket bog. A MANOVA on all fAA’s showed an overall effect of site (*P* < 0.0001), and no main effect of treatment (*P* = 0.5520) but a significant interaction between site and treatment (*P* = 0.0176). The blanket bog had the largest content of total fAA-N (c. threefold larger than the second largest) whereas it had the smallest total soil N content (c. a third of the second smallest); contrastingly, the mesic meadow had the smallest total fAA-N content (c. half the content of the second smallest) in the soil extracts and the largest total soil N content (c. 10% more than in the second largest) (Fig. 1, Table S3).

When comparing fAA-N to the inorganic N forms, fAA-N to NO_3_-N ratio varied by a factor 4 across sites. The fAA-N to NH_4_-N ratio varied by a factor 5 (Table S3); however, no significant difference between sites could be found.

### Redundancy analysis of the free amino acid–vegetation type span

**Fig. 2:**
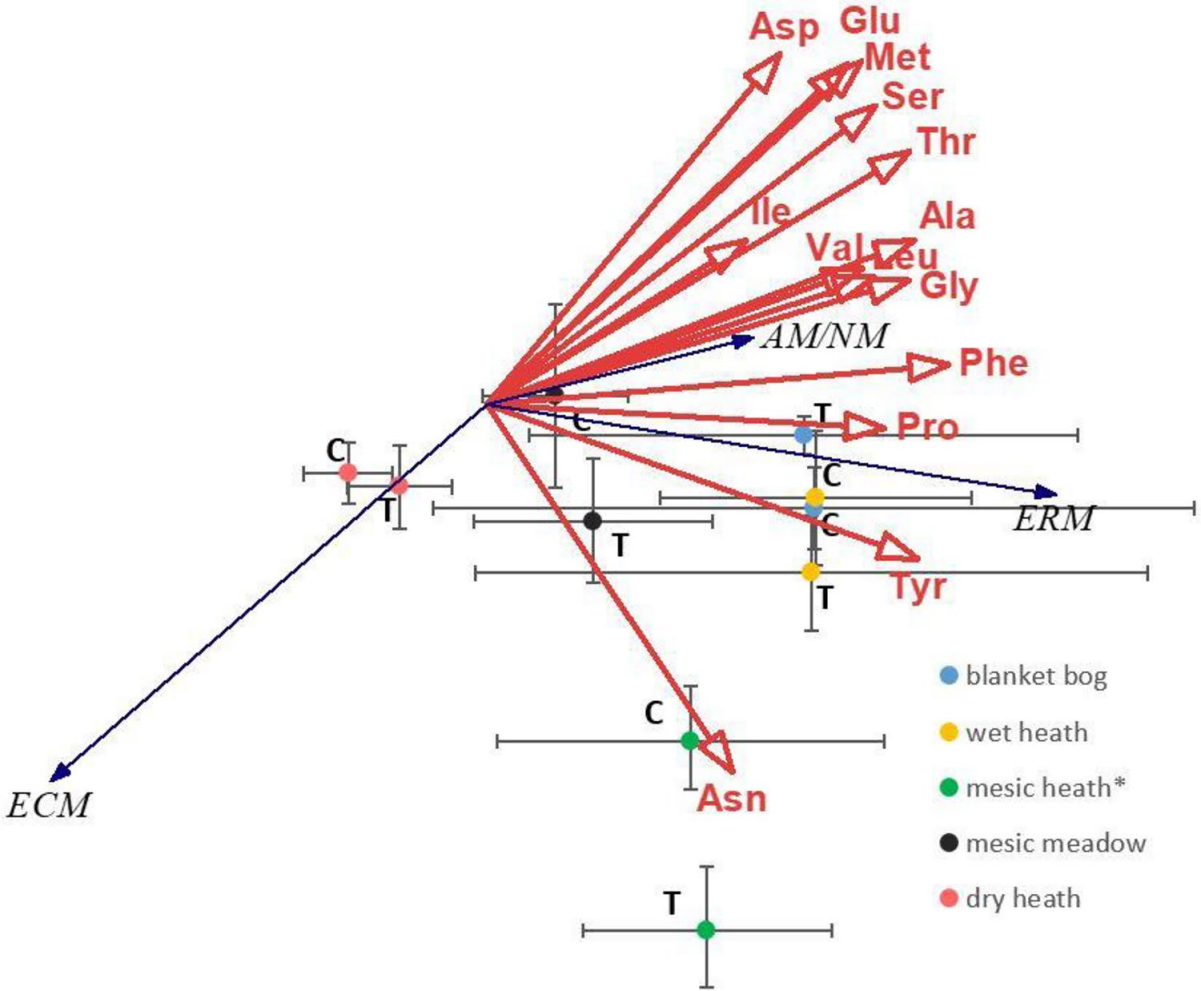
RDA analysis diagram, free amino acid content (µg∙g^−1^ soil; error bars indicate 95% confidence interval). The sites are blanket bog, wet heath, mesic heath, mesic meadow, and dry heath. Treatment T is warming, and C is control (no treatment). The direction of mycorrhizal types ERM, ECM, and AM/NM by black arrows. Total variation is 73.6%, and explanatory variables account for 100.0%, where axis 1 explains 84.2% of the variation and axis 2 explains 11.2% of the variation. Red arrows represent the direction and strength of the individual amino acids: alanine (Ala), glycine (Gly), valine (Val), leucine (Leu), serine (Ser), theonine (The), proline (Pro), asparagine (Asn), aspartate (Asp), methionine (Met), glutamate (Glu), phenylalanine (Phe), tyrosine (Tyr)

The RDA of the individual fAA, constrained by vegetation type as classified by mycorrhizal association, revealed that across all sites, the ECM plants drive the fAA pattern in the opposite direction of the AM/NM and ERM plants (Fig. 2). The higher abundance of AM/NM association at the blanket bog and wet heath coincided with the higher abundance of most of the individual fAA, whereas the higher abundance of ECM associations at the dry and mesic heath had contrastingly lower fAA content. No warming treatment effects were revealed at blanket bog, wet heath, or dry heath. However, at the mesic heath, which had a significant increase in shrubs and decrease of mosses under warming, the RDA analysis separated the warming treatment significantly from control (distinct 95% confidence intervals), driven by changes in vegetation cover and fAA composition jointly (Fig. 2). Similarly for the mesic meadow, which had decreases of AM/NM plants and mosses under warming, a clear separation of warming and control had the same direction as at the mesic heath, with modified amino acid blend and relatively higher cover of ECM and ERM shrubs.

## Discussion

In our study, the fAA content in the heath, meadow, and bog soils of the Scandinavian mountains ranged from 0.5 to 6.2 µg N g^−1^ dry soil, and thus compares well in magnitude with soils from other studies; in the tundra near Toolik Lake, Alaska, the fAA content ranged from 1.6 to 8.3 µg N g^−1^ dry soil, across the ecosystem types wet meadow (lowest), dry heath, shrub tundra and tussock tundra (highest) (Kielland 1995), and in acidic and nonacidic tundra from 1.0 to 4.5 µg N g^−1^ dry soil (Nordin et al. 2004). Even the boreal forests at Fairbanks, Alaska, had fAA content within the same magnitude (0.4 to 4.9 µg N g^−1^ dry soil; Werdin-Pfisterer et al. 2009), and also in a northern Swedish pine forest, the fAA content ranged from 0.8 to 5.2 µg N g^−1^ dry soil (Nordin et al. 2001). The standing pool of fAA is affected by both production (depolymerization) and consumption and by the soils capacity for carrying fAA, i.e., the standing pool of fAA (Homyak et al. 2021), which is controlled by pH, soil organic matter, cation exchange capacity, and clay content. Hence, based on the above, the sites well represent northern latitude soils in fAA abundance and patterns observed below can be generalized.

This study is unique in that we investigated 14 individual free amino acids in the soil in multiple subarctic sites exposed to long-term experimental warming. We found that the negatively charged (acidic) amino acids glutamate and aspartate were the predominant fAA in soil extracts, but qualitative comparisons across to other studies are difficult because most studies only measure a selection of amino acids, due to analytical constraints (Nordin et al. 2001; Werdin-Pfisterer et al. 2009; Warren and Taranto 2010). For example, the dominant amino acids in North American tundra were also glutamate and aspartate as well as arginine, serine, and glycine (Kielland 1995; Nordin et al. 2004). In forest soils, the Northern Swedish pine forest soils had dominance of glutamine and alanine (Nordin et al. 2001), and in North America alanine, asparagine, aspartate, glutamate, glutamine, and histidine dominated (Werdin-Pfisterer et al. 2009). These qualitative differences may thus reflect the vegetation differences.

Field experiments using ^15^N tracing suggest that microbial and plant uptake of glycine (Nordin et al. 2001, 2004; Ma et al. 2015; Ravn et al. 2017), glutamate (Andresen et al. 2008; Gunina et al. 2014; Månsson et al. 2014), aspartate (Schimel and Chapin III 1996, Nordin et al. 2004; Wang et al. 2012), and alanine (Wilkinson et al. 2014) is important in alpine, subarctic, temperate, and boreal ecosystems. On the other hand, fAA are released in soil through depolymerization of peptides which are made available at decomposition. With poorer decomposability of litter from ERM and ECM plants (Cornelissen et al. 2001), the plant abundance affects the fAA balance both through the uptake and through the litter quality. Also in other studies in cold biomes, differences in fAA content have been linked to plant community composition (Feng et al. 2018), with twofold higher fAA concentration in soils with vascular plants compared to moss-dominated soils (Hill et al. 2019). Though the connection of plant type and soil fAA is clear, the causes can be many.

Among our subarctic sites, we found indications that the soil fAA composition was associated with the vegetation and dominance of mycorrhizal types. One possible cause is that the differences in fAA composition and amount across our sites, i.e., largest amount at blanket bog and the composition response to warming at mesic heath (and by tendency at mesic meadow), was driven by a larger uptake of fAA by the ECM and to some extent ERM vegetation (Andresen et al. 2008; Peay 2016; Ravn et al. 2017), as this co-occurred with an increase of ECM and ERM shrubs. However, as the study did not investigate plant uptake, other dynamic mechanisms such as microbial immobilization, or altered plant litter quality, might also have influenced the response. Hypothetically, predicted shrubification of the Arctic (Bjorkman et al. 2018; Scharn et al. 2021) can potentially reduce the fAA content in the soil, and hence lessen the fAA input to N-cycle processes that release inorganic N. This could lead to a positive feedback mechanism enhancing “Arctic greening”—i.e., with increased ERM and ECM shrubs due to increased temperature, their ability to shortcut the N-cycle and take up fAA could result in less substrate input to N mineralization, with consequences for other plant types relying on mineral N (Fig. S2). The mechanism counteracts the temperature-enhanced N turnover by tightening the N cycle via the plant N uptake, and also via slower decomposition of leaf litter dominated by ERM and ECM fungal communities (Cornelissen et al. 2007). Thus, potential increased plant uptake of fAA by plants with ECM (and to some extent ERM) association and their decreased litter decomposition rate can lead to a decrease in the fAA soil content as a side effect of “Arctic greening.”

## Conclusions

The mycorrhizal types influenced the content of individual fAA with a distinct pattern for blanket bog and wet heath together representing vegetation with more AM and non-mycorrhizal-type associations, and this effect was stronger than the effect of the warming treatment. The findings suggest that lower soil fAA contents can be driven by a larger uptake of fAA by the ECM (and to some extent ERM) vegetation, and by altered litter quality and decomposition and depolymerization of organic compounds in sites dominated by these plant functional groups. We suggest that vegetation changes such as shrubification may indirectly reduce the fAA soil content and influence its composition. This fAA retention in the ecosystem serves as retention of N within a tight soil–mycorrhiza–plant cycle, which reduces potential leaching losses of inorganic N. We suggest that such shrubification helps to retain N within these ecosystems.

## Supplementary Information

Below is the link to the electronic supplementary material.

Supplementary file1 (DOCX 363 KB)

Supplementary file2 (DOCX 49 KB)

Supplementary file3 (DOCX 38 KB)

Supplementary file4 (DOCX 82 KB)

## Data Availability

Data will be available by contacting the first author.
