# Supplementary material for: Patterns of free amino acids in tundra soils reflect mycorrhizal type, shrubification, and warming
Source: Mycorrhiza. 2022 Mar 21;32(3-4):305–13. doi: 10.1007/s00572-022-01075-4 (PMC9184409; doi:10.1007/s00572-022-01075-4)

## Supplementary material

**Supplementary methods description**

Gravimetric soil moisture content was determined on c.a. 10 g of soil (24 hours at 100 °C). Soil organic matter was determined on c.a. 2 g of dried soil samples by loss of ignition (4 hours at 500° C). Total N and C were determined with an elemental analyser at Isogot (GSL Sercon Ltd., Crewe, UK).

To determine microbial biomass 1.5 mL of CHCl_3_ was added directly to a subsample of 10 g soil and this was kept airtight in darkness for 2 days inside a fume hood, then extracted with 50 mL 2 mol∙L^-1^ KCl (140 rd min^-1^ for 30 minutes). In parallel another subsample of 10 g fresh soil was extracted. Supernatant of the soil suspension was filtered through GF/D Whatman filters and frozen until analysis was performed (Perakis 2005). To determine total N in liquid extracts all present N forms were converted to NO_3_^-^ with persulfate to be analysed. NO_3_^-^ and NH_4_^+^ concentration were determined using flow injection colorimetric analysis (FIAstar 5000 Analyser). Dissolved organic carbon (DOC) content of extracts were determined using TOC analyser (TOC 5000A, Shimadzu), in the laboratory at University of Copenhagen (Denmark). The priority during field work was sampling of soils for fresh extraction of free amino acids and soil samples for KCl extraction had only minimal replication of 2-6 per treatment and site. Furthermore, we did not retrieve KCl extracts at the mesic meadow, but published data on ammonium and nitrate in 2004 (Björk et al. 2007a) was used by rescaling with the SOM content from 2013 (Table 1).

Microbial N (MicN) was calculated as the difference of total N in extracts (TDN) with and without CHCl_3_ incubation, divided by the proportion of extractability (K_EN_ = 0.40; (Joergensen 1996b)): MicN = (TDN_CHCl3_ – TDN) / K_EN_

Microbial C (MicC) was calculated as the difference of total C in extracts (DOC) with and without CHCl_3_ divided by the proportion of extractability (K_EC_ = 0.45; (Joergensen 1996a)): MicC = (DOC_CHCl3_ – DOC) / K_EC_

**Table S1 (heading)**. Site characteristics of the five locations in the Scandinavian mountain range the altitude in meters above sea level (m.a.s.l.); mean annual temperature (MAT); mean annual precipitation (MAP); given the dominant plant species of the plots. The year in which the OTC experiments were initiated, and the dimension and material of the open top chambers (OTC) is presented. The OTC treatment effect on temperature increase in air and soil (measured 5 cm above and below surface), the period of the OTC treatment, the number of replicates, and the date and year of the soil sampling and year of vegetation survey in brackets, are presented.

**Table S2 (heading)**. Log response ratio LRR (equation 3) of the warming treatment relative to control for the soil variables and plant growth form. LRR calculated for dissolved organic carbon (DOC), microbial carbon (MicC), microbial nitrogen (MicN), nitrate (NO_3_), ammonium (NH_4_) and microbial C to N ratio and free amino acid nitrogen (fAA-N) and carbon (fAA-C) content and plant cover in groups of mycorrhiza type: Arbuscular and non-mycorrhizal together as AM/NM; Ericoid mycorrhiza species (ERM), Ecto mycorrhiza species (ECM) and in groups of plant growth form (forbs, graminoids, shrubs and mosses). Bold indicates significance (P < 0.05) and italics at statistical tendency (P < 0.1) towards an effect of treatment from the one-way ANOVA, see text. Not determined is indicated by nd.

**Table S3.**

### Soil properties

The MANOVA on dissolved and microbial soil C and N showed an overall effect of site (P<0.0001), treatment (P=0.0002) and the interaction site×treatment (P=0.0015). Total soil N (ranging av. 0.6 to 2.1 %), gravimetric soil water content (ranging av. 121 to 703 %) and SOM (ranging av. 31 to 98 %) were different between sites (all P < 0.0001), but were not affected by warming and there were no interactions (Table S2). For DOC (varying 10-fold) there were effects of site (P < 0.0001), treatment (P = 0.0002) and the interaction (P = 0.0008; Table S2). Microbial C (varying two-fold) tended to differ between sites (P = 0.067) and for microbial N (varying three-fold) there was a significant effect of site (P = 0.036), an interaction between site and treatment (P = 0.013) and a tendency of treatment effect (P = 0.057; Table S2). Within sites, warming doubled microbial N at the wet heath (P = 0.014). NH_4_^+^ tended to increase under warming in the blanket bog (doubled) (P = 0.062) and quadrupled in the wet heath (P = 0.064). The blanket bog had higher total soil and microbial C to N ratios, higher SOM content and significantly higher DOC concentration and water content as compared to all other sites (Table S2). The dry heath had the smallest SOM, DOC and microbial N, and also the lowest soil C to N ratio (Table S2).

**Table S3 (heading).** Soil characteristics of the sites in 2013. Treatment T is warming, C is control (no treatment). Data represent average of replicate treatments with standard errors in brackets. P-values of significant (< 0.05) effects of site, treatment and the interaction from two way ANOVA, *** indicates P < 0.001; * indicates P < 0.05. Bold letters A, B or C indicate significant differences between sites. SOM is soil organic matter by loss on ignition, gravimetric water content, total N and C are determined by elemental analysis. Nitrate (NO_3_), ammonium (NH_4_), total dissolved N (TDN), microbial N (MicN), dissolved organic C (DOC) and microbial C (MicC) were measured in 2 mol∙L^-1^ KCl extracts; free amino acid (fAA) content determined based on 10 mmol∙L^-1^ CaSO_4_ extracts; (dates and site details in Table 1). Not determined is indicated by nd. Data in italics from mesic meadow are obtained from Björk *et al.* 2007.

**Supplementary Figures**

**Figure S1**. Concentrations of individual measured free amino acids in the soil extracts at the blanket bog (A), the wet heath (B), the mesic heath (C), the mesic meadow (D) and the dry heath (E). Treatment T is warming, C is control (no treatment).

Figure S1


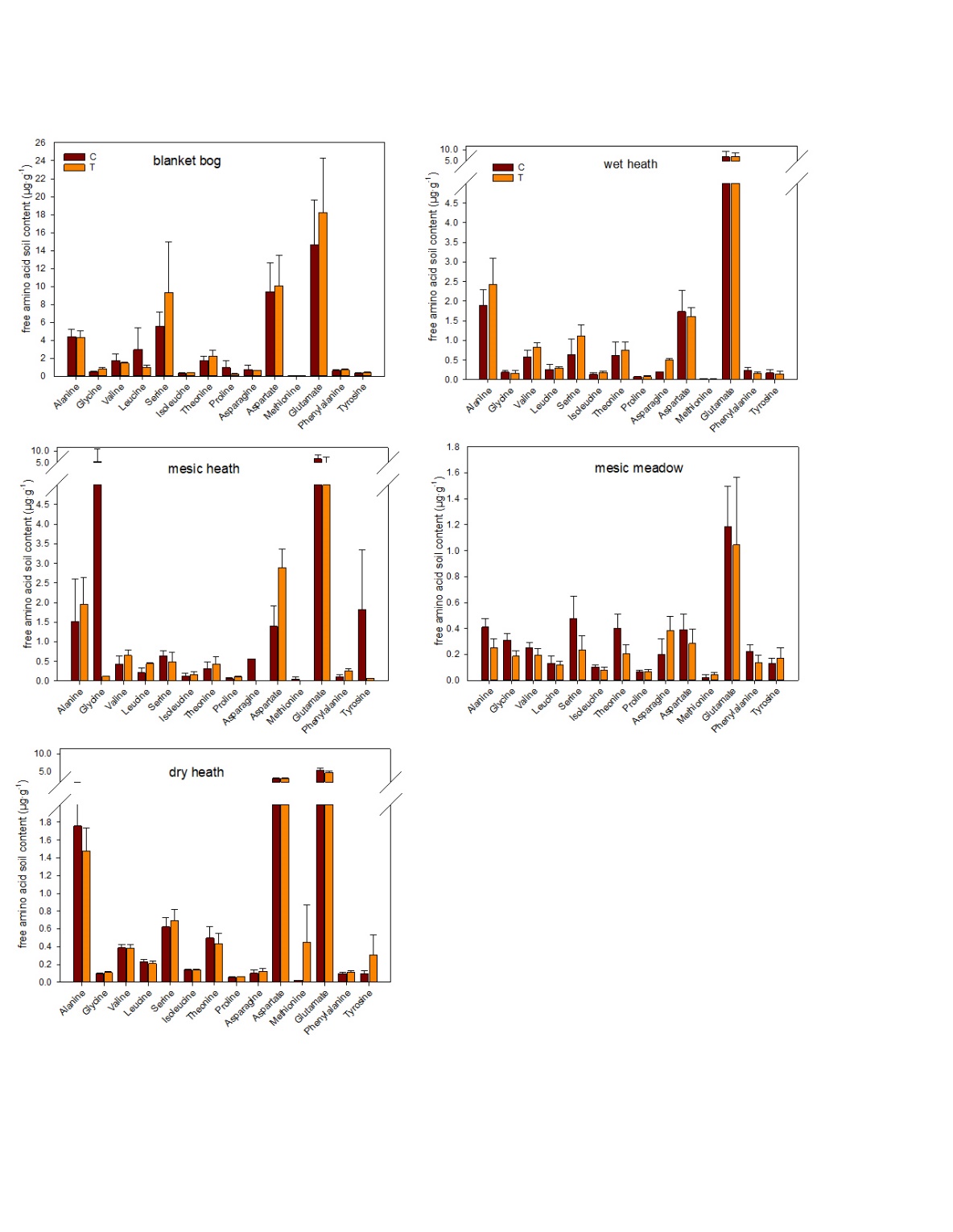


**Figure S2.** Conceptual diagram for a hypothetical alternative mechanism behind arctic greening. The pools and processes respond to the factors i. presence of **EMYCO** (jointly ERM (ericoid) / ECM (ecto) mycorrhiza) and ii. climatic warming **W**; **+** indicates a positive response and **–** indicates a negative response. The diagram illustrates mechanisms affecting the pools: soil fAA (free amino acids), the biomass of ERM + ECM (the biomass causing Arctic greening through shrubification) and of AM (arbuscular mycorrhizal) vegetation, plant litter and IN (inorganic nitrogen) and the fluxes: litter decomposition rate, (gross) mineralization rate, and the plant uptake of N from fAA and IN. ECM+ERM plant biomass responds positive to warming, why their litter pool also respond positive in amount, and the litter decomposition rate responds positive to warming, why fAA pool theoretically could increase. However, the ECM+ERM uptake of fAA is increased due to their increased biomass, why the fAA pool decreases in response to EMYCO. Furthermore, litter decomposition rate of ECM+ERM litter is decreased, also giving fAA pool less input, also giving a negative response to EMYCO. The gross N mineralization rate can decrease as consequence of decreased fAA availability for the mineralization (or increase if the indirect or a direct response to W rules). A decrease in mineralization causes decrease in the IN pool, which causes decreased uptake of IN in AM/NM plants. The AM/NM plants can therefore respond with decreased biomass due to the decrease in the preferred IN. As the ECM+ERM plant biomasses have increased uptake of fAA, and at the same time positive response under warming they respond positively and can expand causing Arctic greening.

Figure S2


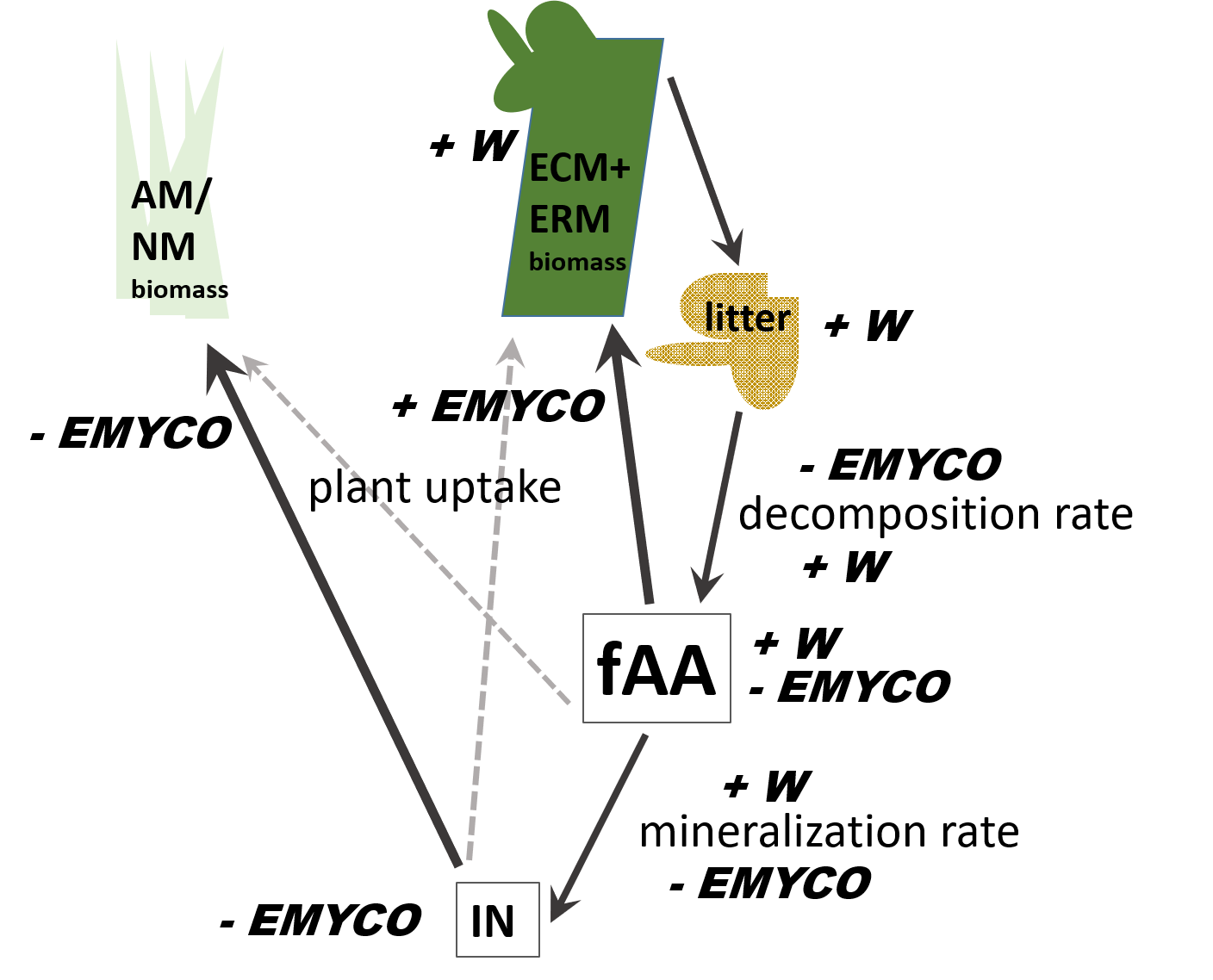

Supplement: Supplementary file 1 — Supplementary file1 (DOCX 363 KB) [file 572_2022_1075_MOESM1_ESM.docx]
