# Supplementary material for: Patterns of free amino acids in tundra soils reflect mycorrhizal type, shrubification, and warming
Source: Mycorrhiza. 2022 Mar 21;32(3-4):305–13. doi: 10.1007/s00572-022-01075-4 (PMC9184409; doi:10.1007/s00572-022-01075-4)
Supplement: Supplementary file 2 — Supplementary file2 (DOCX 49 KB) [file 572_2022_1075_MOESM2_ESM.docx]

| **Site** | **Altitude (m.a.s.l.)** | **MAT**  **(**°**C)** | **MAP (mm)** | **Dominant species** | **OTC Since** | **OTC dimension (m)** | **OTC material** | **OTC treatment** | **Time period of warming and reps.** | **Sample dates** | **Reference** |
| --- | --- | --- | --- | --- | --- | --- | --- | --- | --- | --- | --- |
| **blanket bog**  ***(Abisko, shore of Torneträsk)*** | 350 | 0.2 | 340 | *Empetrum nigrum* | 2000 | Hexagon:  diagonal: 2.2  height: 0.50 | plexiglass | Air: +1°C  Soil: +1°C | All year (5 repl.) | 30. July 2013  (2013) | *Dorrepaal at al. 2009.* |
| **wet heath**  ***(Abisko, near Torneträsk)*** | 400 | 0.2 | 340 | *Empetrum nigrum;*  *Andromeda polifolia* | 1999 | Quadratic tent:  diagonal: 1.2; height 0.5 | polyethylene | Air: +3 to 4 °C  Soil: +1 °C | Summer (6 repl.) | 27. August 2013  (2011) | *Finderup Nielsen et al. 2019;*  *Rinnan et al. 2007; Lett and Michelsen 2014* |
| **mesic heath**  ***(Abisko)*** | 450 | 0.2 | 340 | *Casssiope tetragona; Betula nana* | 1989 | Quadratic tent:  diagonal: 1.2; height 0.5 | polyethylene | Air: +1 to 3 °C  Soil: +1 °C | Summer (6 repl.) | 28. August 2013  (2014) | *Michelsen et al. 2012; Sorensen et al. 2011* |
| **mesic meadow**  ***(Latnajaure, Abisko)*** | 988 | 1.7 | 855 | *Vaccinium vitis-idaea; Dryas octopetala* | 1993 | Hexagon:  side: 0.38 × 0.40 × 0.57; height: 0.35 | plexiglass | Air: +1.6 °C  Soil: +0.3 | All year (10 repl.) | 3. August 2013 (2016) | *Marion 1997* |
| **dry heath**  ***(Finse)*** | 1554 | -2.1 | 1030 | *Dryas octopetala* | 2000 | Octagon:  side: 0.38 × 0.40 × 0.57; height: 0.35 | plexiglass | Air: +1.5 °C  Soil: +1 °C | All year (40 repl.) | 12. July 2013  (2011) | *Klanderud and Totland 2007, 2005* |

**Table S1**. Site characteristics of the five locations in the Scandinavian mountain range the altitude in meters above sea level (m.a.s.l.); mean annual temperature (MAT); mean annual precipitation (MAP); given the dominant plant species of the plots. The year in which the OTC experiments were initiated, and the dimension and material of the open top chambers (OTC) is presented. The OTC treatment effect on temperature increase in air and soil (measured 5 cm above and below surface), the period of the OTC treatment, the number of replicates, and the date and year of the soil sampling and year of vegetation survey in brackets, are presented.
