# Supplementary material for: Patterns of free amino acids in tundra soils reflect mycorrhizal type, shrubification, and warming
Source: Mycorrhiza. 2022 Mar 21;32(3-4):305–13. doi: 10.1007/s00572-022-01075-4 (PMC9184409; doi:10.1007/s00572-022-01075-4)
Supplement: Supplementary file 3 — Supplementary file3 (DOCX 38 KB) [file 572_2022_1075_MOESM3_ESM.docx]

**Table S2**. Log response ratio LRR (equation 3) of the warming treatment relative to control for the soil variables and plant growth form. LRR calculated for dissolved organic carbon (DOC), microbial carbon (MicC), microbial nitrogen (MicN), nitrate (NO_3_), ammonium (NH_4_) and microbial C to N ratio and free amino acid nitrogen (fAA-N) and carbon (fAA-C) content and plant cover in groups of mycorrhiza type: Arbuscular and non-mycorrhizal together as AM/NM; Ericoid mycorrhiza species (ERM), Ecto mycorrhiza species (ECM) and in groups of plant growth form (forbs, graminoids, shrubs and mosses). Bold indicates significance (P < 0.05) and italics at statistical tendency (P < 0.1) towards an effect of treatment from the one-way ANOVA, see text. Not determined is indicated by nd.

| **Site** | **DOC** | **MicC** | **NO_3_** | **NH_4_** | **MicN** | **Mic C to N ratio** | **FAA-N** | **FAA-C** | **AM/NM** | **ERM** | **ECM** | **Forbs** | **Graminoids** | **Shrubs** | **Mosses** |
| --- | --- | --- | --- | --- | --- | --- | --- | --- | --- | --- | --- | --- | --- | --- | --- |
| **Blanket bog** | 0.9 | -0.1 | 0.2 | ***0.5*** | 0.3 | -0.4 | 0.1 | -0.4 | -0.1 | -0.3 | -0.7 | -0.4 | ***1.0*** | -0.3 | 0.0 |
| **Wet heath** | 0.9 | 0.7 | 0.2 | ***1.3*** | **0.9** | -0.2 | 0.2 | 0.2 | 0.4 | 0.0 | 0.4 | 1.0 | -0.1 | 0.1 | 0.5 |
| **Mesic heath** | 0.0 | -0.2 | 0.1 | 0.0 | -0.1 | -0.1 | -0.3 | -0.2 | -0.1 | 0.7 | 0.5 | 0.0 | -0.5 | **0.6** | **-2.2** |
| **Mesic meadow** | nd | nd | nd | nd | nd | nd | -0.4 | -0.5 | ***-0.4*** | 0.4 | 0.2 | -0.3 | -0.4 | 0.6 | **-0.7** |
| **Dry heath** | 0.0 | -0.2 | -0.2 | 0.2 | -0.1 | 0.0 | 0.0 | 0.0 | 0.2 | 1.0 | **-0.2** | 0.1 | 0.2 | -0.2 | 0.1 |
