# Supplementary material for: Patterns of free amino acids in tundra soils reflect mycorrhizal type, shrubification, and warming
Source: Mycorrhiza. 2022 Mar 21;32(3-4):305–13. doi: 10.1007/s00572-022-01075-4 (PMC9184409; doi:10.1007/s00572-022-01075-4)
Supplement: Supplementary file 4 — Supplementary file4 (DOCX 82 KB) [file 572_2022_1075_MOESM4_ESM.docx]

**Table S3.** Soil characteristics of the sites in 2013. Treatment T is warming, C is control (no treatment). Data represent average of replicate treatments with standard errors in brackets. P-values of significant (< 0.05) effects of site, treatment and the interaction from two way ANOVA, *** indicates P < 0.001; * indicates P < 0.05. Bold letters A, B or C indicate significant differences between sites. SOM is soil organic matter by loss on ignition, gravimetric water content, total N and C are determined by elemental analysis. Nitrate (NO_3_), ammonium (NH_4_), total dissolved N (TDN), microbial N (MicN), dissolved organic C (DOC) and microbial C (MicC) were measured in 2 mol∙L^-1^ KCl extracts; free amino acid (fAA) content determined based on 10 mmol∙L^-1^ CaSO_4_ extracts; (dates and site details in Table 1). Not determined is indicated by nd. Data in italics from mesic meadow are obtained from Björk *et al.* 2007.

| **Site** | **Treat-ment** | **SOM (%)** | | **Gravimetric water content (%)** | | **Total soil N (%)** | | **NO_3_ µg N g^-1^ soil** | **NH_4_ µg N g^-1^ soil** | **fAA-N μg N g^-1^ soil** | | **TDN µg N g^-1^ soil** | **MicN µg N g^-1^ soil** | | **DOC μg C g^-1^ soil** | | **MicC μg C g^-1^ soil** | **C/N** | **fAA-N / NO_3_-N** | **fAA-N/ NH_4_ -N** | **fAA-C/ DOC** | **Mic C/N** |
| --- | --- | --- | --- | --- | --- | --- | --- | --- | --- | --- | --- | --- | --- | --- | --- | --- | --- | --- | --- | --- | --- | --- |
| **Blanket bog** | C | 98.0 (1.0) | A | 599  (192) | A | 0.64 (0.07) | B | 2.62 (0.63) | 6.80 (0.43) | 5.42 (1.29) | A | nd | 371 (98) | B | 989 (72) | A | 5950 (942) | 68.53 | 2.07 | 0.80 | 0.031 | 16.0 |
|  | T | 96.5 (0.5) |  | 703  (146) |  | 0.55 (0.06) |  | 3.22 (0.59) | 11.76  (1.22) | 6.12 (1.64) |  | 10.8 | 480 (48) |  | 2535 (637) |  | 5279  (249) | 75.51 | 1.90 | 0.52 | 0.008 | 11.0 |
| **Wet heath** | C | 67.8 (6.4) | AB | 301 (31) | AB | 1.54 (0.10) | A | 2.40 (0.28) | 17.87  (4.23) | 1.38 (0.46) | B | 5.0 (0.7) | 430 (58) | A | 492 (61) | B | 3689 (24) | 26.99 | 0.58 | 0.08 | 0.010 | 8.6 |
|  | T | 83.9 (8.0) |  | 589  (86) |  | 1.43 (0.10) |  | 2.99 (0.29) | 65.84 (12.61) | 1.74 (0.15) |  | 17.1 (4.5) | 1051 (85) |  | 1153 (255) |  | 7749  (575) | 28.47 | 0.58 | 0.03 | 0.005 | 7.4 |
| **Mesic heath** | C | 72.3 (7.6) | B | 175 (21) | CD | 1.75 (0.15) | A | 1.38 (0.24) | 29.30 (13.39) | 1.91 (1.08) | B | 21.4 (5.4) | 593 (88) | AB | 825 (150) | B | 6452  (1144) | 23.15 | 1.38 | 0.07 | 0.007 | 10.9 |
|  | T | 75.2 (9.1) |  | 166  (12) |  | 1.79 (0.20) |  | 1.46 (0.11) | 29.52 (10.74) | 1.42 (0.42) |  | 19.0  (4.4) | 527 (44) |  | 817 (141) |  | 5088  (389) | 19.40 | 0.97 | 0.05 | 0.006 | 9.7 |
| **Mesic meadow** | C | 70.8 (4.1) |  | 255 (14) | BC | 2.07 (0.07) | A | *0.9* | *24* | 0.70 (0.16) | B | nd | nd |  | nd |  | nd | 17.42 | *0.8* | *0.03* | nd | nd |
|  | T | 64.6 (4.6) |  | 210  (18) |  | 1.85 (0.09) |  | nd | nd | 0.49 (0.13) |  | nd | nd |  | nd |  | nd | 18.33 | nd | nd | nd | nd |
| **Dry heath** | C | 35.0 (4.1) | C | 121 (20) | D | 1.42 (0.09) | A | 2.57 (0.94) | 34.74 (4.82) | 1.35 (0.13) | B | 11.1 (1.7) | 482 (44) | B | 244 (39) | C | 3953  (593) | 15.16 | 0.53 | 0.04 | 0.019 | 8.2 |
|  | T | 31.2 (8.6) |  | 121  (38) |  | 1.56 (0.10) |  | 2.09 (0.53) | 42.13 (15.54) | 1.36 (0.14) |  | 13.3  (5.5) | 421 (108) |  | 250 (69) |  | 3387  (1085) | 16.37 | 0.65 | 0.03 | 0.018 | 8.1 |
| *Site*  *Treatment*  *Treatment*site* |  | ***  n.s.  n.s. | | ***  n.s.  n.s. | | ***  n.s.  n.s. | | n.s.  n.s.  n.s. | n.s.  n.s.  n.s. | ***  n.s.  n.s. | | n.s.  n.s.  n.s. | *  n.s.  * | | ***  ***  *** | | n.s.  n.s.  n.s. |  |  |  |  |  |
